# The mitochondrial lineage U8a reveals a Paleolithic settlement in the Basque country

**DOI:** 10.1186/1471-2164-7-124

**Published:** 2006-05-23

**Authors:** Ana M González, Oscar García, José M Larruga, Vicente M Cabrera

**Affiliations:** 1 Department of Genetics, Faculty of Biology, University of La Laguna, 38271 Tenerife, Canary Islands, Spain; 2 Area de Lab. Ertzaintza, Universidad del País Vasco, Larrauri Mendotxe Bidea 18, 48950, Erandio, Bizkaia, Spain

## Abstract

**Background:**

It is customary, in population genetics studies, to consider Basques as the direct descendants of the Paleolithic Europeans. However, until now there has been no irrefutable genetic proof to support this supposition. Even studies based on mitochondrial DNA (mtDNA), an ideal molecule for constructing datable maternal genealogies, have failed to achieve this. It could be that incoming gene flow has replaced the Basque ancient lineages but it could also be that these lineages have not been detected due to a lack of resolution of the Basque mtDNA genealogies. To assess this possibility we analyzed here the mtDNA of a large sample of autochthonous Basques using mtDNA genomic sequencing for those lineages that could not be unequivocally classified by diagnostic RFLP analysis and control region (HVSI and HVSII) sequencing.

**Results:**

We show that Basques have the most ancestral phylogeny in Europe for the rare mitochondrial subhaplogroup U8a. Divergence times situate the Basque origin of this lineage in the Upper Palaeolithic. Most probably, their primitive founders came from West Asia. The lack of U8a lineages in Africa points to an European and not a North African route of entrance. Phylogeographic analysis suggest that U8a had two expansion periods in Europe, the first, from a south-western area including the Iberian peninsula and Mediterranean France before 30,000 years ago, and the second, from Central Europe around 15,000–10,000 years ago.

**Conclusion:**

It has been demonstrated, for the first time, that Basques show the oldest lineages in Europe for subhaplogroup U8a. Coalescence times for these lineages suggest their presence in the Basque country since the Upper Paleolithic. The European U8 phylogeography is congruent with the supposition that Basques could have participated in demographic re-expansions to repopulate central Europe in the last interglacial periods.

## Background

Considering Basques as the direct descendants of Paleolithic Europeans has become a multidisciplinary premise. However, there is no irrefutable evidence for this supposition. The Basque country has a well represented archaeological record in Paleolithic and Mesolithic periods [1] but Archaeology can seldom differentiate an "in situ" cultural evolution from successive waves of new incomers. Basques speak a non Indo-European language with no close affinities with any other extant language but, even if their roots could be found, they would not reach the Paleolithic deepness due to the fast rate of change of languages. Classical population genetic studies, showed the Basques as one of the major outliers in Europe [2]. Nevertheless, these results can be explained by genetic drift which implies isolation but not necessarily an old history for that population. Lack of recombination and the fast mutation rate made mtDNA the ideal molecule to construct maternal genealogies, which frame in time and space the evolution and dispersion of human populations. However, until now, mtDNA studies on the Basques have only confirmed its low genetic diversity in a common Western Europe background [3-5]. It has been proposed, on the basis of their geographic distributions, that several mitochondrial lineages as V [6,7], and the H1 and H3 subgroups [4,5,8] are markers of a Paleolithic human dispersal from southwestern Europe, including the Basque country, to Northeast Europe. However, diversities for these lineages are not higher in Basques than in Central Europeans. It could be possible that this lack of distinctness in Basques is real, in fact, even small levels of gene flow during enough time might have replaced the majority of their ancient lineages [9], but it could also be possible that this uniformity is due to a lack of resolution of the Basque mtDNA genealogies [10].

To deal with this possibility, we analyzed a sample of 211 unrelated Basques using the hypervariable segment of the mtDNA control region (HVSI-II) and diagnostic RFLP analysis, and sequenced the complete mitochondrial DNA of the rare lineages.

## Results and discussion

**Figure 1:**
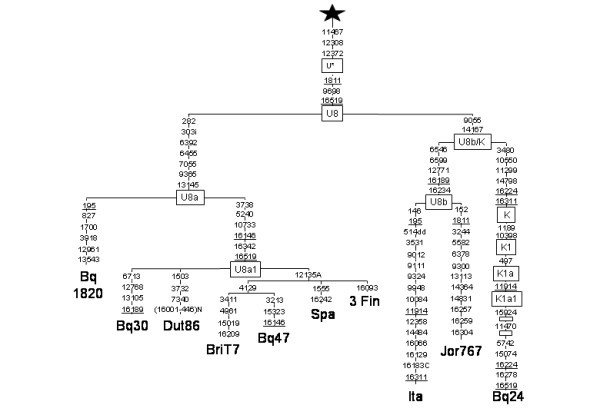
**Phylogenetic tree based on complete U8 sequences**. Numbers along links refer to nucleotide positions. Open boxes are nodes from which other (not shown) sequences branch. A, C, indicate transversions; "d" deletions and "i" insertions. Star has the following mutations with respect to rCRS: 73, 263, 311i, 750, 1438, 2706, 4769, 7028, 8860, 11719, 14766 and 15326 and the following ones respect to L3*: 8701, 9540, 10398, 10873, 12705, 15301, 16223, 16519. Subject origins are: Dutch (Dut86; [38]), Spanish (Spa) and Italian (Ita) [20], 3 Finns (Fin; [14]). British-Australian (BriT7; Obayashi T, Tanaka M, personal communication), Jordan (Jor767) and 4 Basques (Bq 1820, 30, 47, 24)

**Figure 2:**
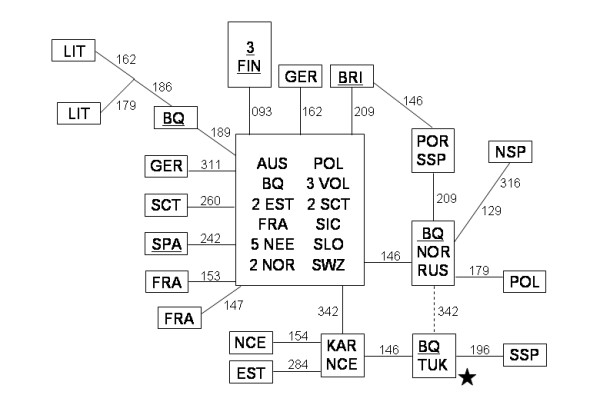
**Reduced median network relating U8a HVSI/II sequences**. Star is CRS for HVSI and 73, 282 for HVSII. Numbers along links refer to nucleotide positions minus 16000; Underlined subjects were complete sequenced. The broken line is the less probable link in accordance with completed sequences (Fig.1). Codes are: AUS = Austrian; BQ = Basque; BRI = British-australian; EST = Estonian; FIN = Finn; FRA = French; GER = German; KAR = Karelian; LIT = Lithuanian; NCE = North-central European; NEE = Northeast European; NOR = Norwegian; NSP = Northeast Spanish; POL = Polish; POR = Portuguese; RUS = Russian; SCT = Scotch; SIC = Sicilian; SLO = Slovenian; SPA = Spanish; SSP = South Spanish; SWZ = Swiss; TUK = Turk; VOL = Volga-Ural.

The analysis of the Basque sample showed three haplotypes (CRS, 16342, and 16278 16311) that by their mutated positions in the control region had uncertain subhaplogroup adscription, but that by diagnostic RFLPs (+12308 Hinf I) belonged to haplogroup U/K. Also, we found one individual (16146 16189 16342) that belongs to the scarce U8a subhaplogroup. Complete sequencing of the four rare U haplotypes and their inclusion in a phylogenetic tree [11] with all published U complete sequences (data not shown) allowed their correct subhaplogroup affiliation. A more schematic tree (Fig.1) shows that one of the lineages (Bq24) belongs to subhaplogroup K1a1 [12], being a back-mutation of the diagnostic position 16224 its main peculiarity. Its most related K1a1 complete sequences are the eleven found by Herrnstadt et al. [13] and Finn 153 in Finnilä et al. [14]. Applying a mutation-rate of 1.26 × 10^-8 ^[15] to their average sequence divergence [16] a radiation upper bound of 12 ± 4 Ky is obtained for this group. The other three lineages clustered into the rare subhaplogroup U8a [17]. This subhaplogroup can be RFLP diagnosed as -7055 Alu I. What is outstanding of these sequences is their great genetic diversity that extends the range of all known U8a European sequences (Fig.1). This Basque diversity specially contrasts with the lack of variation in the Finn sequences. Furthermore, the phylogenetic radiation of their U8a lineages (Fig. 1 and 2) is characteristic of an old population without recent exponential growth [18]. In fact, the most ancestral sequence (Bq1820) indicates that U8a lineages could have been in the Basque country since 28 ± 9 Ky, and that the other Basque lineages, belonging to the U8a1 subgroup (RFLP diagnosed as -3737 Hph I and + 5235 MspA1 I), participated in a more recent European expansion around 13 ± 5 Ky, similar to that estimated for K1a, and congruent with a re-expansion from an Iberian refuge when glaciers retreated in Europe proposed for other mtDNA clades [4,5,7]. Although all the U8a complete sequences belong to Europeans, the ancestral radiation of haplogroup U most probably occurred in western Asia shortly after the out of Africa episode [19], with early branch expansions to India (U2), Europe (U5) and North Africa (U6). U8 may be considered another main branch with a broad geographic range. Its first split separated U8a from U8b/K around 57 ± 11 Ky. Relatively short in time a new subdivision gave the sister clades U8b and K [17]. Until now there was only one completely sequenced U8b subject [20]. The addition of our Jordan 767 sequence to the tree (Fig. 1) gives a branching point for U8b, defined by transitions 6546, 6599 and 12771. Two of them, 6546 and 12771, can be MnlI-RFLP-detected. Curiously, the similar number of substitutions to the coalescence point of the three U8 branches U8a (5), U8b(5), K(6), suggests that all of them radiated at a similar age, supporting the hypothesis that, most probably, global climatic changes favored human expansions simultaneously at a continental scale [21]. When only RFLPs and/or partial sequence data are available, U8a haplotypes in general can be identified by the -7055 Alu I RFLP or the 73, 282 HVSII motif, and the majority of U8a1 derivates in particular by the16146, 16342 HVSI motif. In turn, U8b can be classified by the 16189, 16234 HVSI motif. From a total of 20,563 sequences studied 10,677 could be, unequivocally, U8a analyzed (see Additional file 1) and referenced (see Additional file 2). For the remaining (9,886), in general, only their U8a1 assignation was possible. Analysis of 19,133 Eurasian, and 1,430 North African published and unpublished RFLP/HVSI/HVSII sequences showed a scattered but widespread U8a/1 distribution that is restricted to Europe. Its frequency (See Additional file 1 for the European distribution of subhaplogroup U8a, and Additional file 2 for references) ranges from 0 in the majority of samples to 8% (although with a 95% coefficient range around 2.9–21.4) in the region of Var in Southeast France [22]. In spite of its moderate sample size an important characteristic of this region is its high U8a polymorphism as all the three lineages detected are different. The U8a eastern boundaries seem to be in the Volga region near the Urals [23]. U8b is also a quantitatively minor clade that partially overlaps with U8a in Europe. However, its presence in the Caucasus [24], Iran [17], the Near East [25], and North Africa [26], where U8a has not been detected, attests a more southern geographic distribution. The third sister clade K, is the most widespread and abundant covering the U8a and U8b ranges [24] and even reaching India [27]. A network [28] built with the 48 U8a sequences found (Fig. 2), could be rooted and resolved attending to the phylogeny of the U8 complete sequences. Its most ancestral node is represented by Bq1820 and the only Anatolian lineage assignable to haplogroup U8a, both carrying the CRS motif in HVSI, and transitions 73, 282 in HVSII. This ancient connection might trace the hypothetic route followed by the U8a ancestor from West Asia to the Basque country. The absence of U8a in North-Africa and its extremely rare presence in the eastern Mediterranean area further reinforces this continental route of entrance against a southern alternative. It is deduced from the network (Fig. 2) that a first U8a radiation in Europe affected Iberia, Central Europe and reached the Baltic. A second, U8a1, broader expansion further enlarged its range to Russia and Scotland where the U8a diversities are lower than in the central area (Table 1). As the total rooted network does not achieve the star genealogy, to calculate coalescence ages [29], we estimated the average distance and coalescence age to the U8a1 founder haplotype (16146 16342) and to the ancestral U8a haplotype (CRS) independently, representing, in the last case, the U8a1 radiation by only one basic lineage. Time estimations for the younger U8a1 expansion was 14 ± 5 Ky and 23 ± 14 Ky for the U8a subset that, if added to first, would give a total time for the U8a coalescence of around 37 ± 14 Ky. Notice that both HVSI estimations are higher than, but not significantly different, from those calculated using complete sequences. To compare the U8a diversity (p ± σ) among regions, we grouped the European populations in different Paleolithic areas [24,30]. The greatest diversity was found in the Iberian Peninsula when Basques are included, followed by the North Central area (Table 1). These data agree with the primary and secondary origins of expansion proposed on phylogenetic grounds, weakening the possibility that Basques would have obtain their total U8a diversity through recent immigrations from other European areas and reinforcing the hypothesis that the first U8a radiation in Europe happened in an area in which the Basque country was included.

**Table 1:** Gene diversity (p) and frequency of U8a/1 in different European macro-regions.

Region^a^	n	p ± σ	sample	U8a (%)
BQ	4	2.0 ± 1.7	367	1.1
IP	9	2.8 ± 1.9	3173	0.3
MC	1		1471	0.1
ME	1		885	0.1
AN	1		620	0.2
CA	0		1681	-
SW	3	1.3 ± 1.4	109	2.8
AL	2	0.0 ± 0.0	766	0.3
SE	0		234	-
NW	3	0.7 ± 0.8	2588	0.1
NC	6	2.2 ± 1.6	2975	0.2
NE	15	1.6 ± 1.1	2320	0.6
VU	3	0.0 ± 0.0	1125	0.3
SC	3	0.0 ± 0.0	1186	0.3

^a ^Name region: BQ = Basque; IP = Iberian Peninsula; MC = Mediterranean Centre; ME = Mediterranean East; AN = Anatolia; CA = Caucasus; SW = Southwest Europe; AL = Alpine; SE = Southeast Europe; NW = Northwest Europe; NC = North central Europe; NE = Northeast Europe; VU = Volga-Ural; SC = Scandinavia

## Conclusion

In summary, the analysis of U8a lineages supports the idea that Basques have lived in their country since the Paleolithic, and that they could have participated in demographic re-expansions to repopulate central Europe in the last interglacial periods. Furthermore, these primitive U8a founders most probably reached the Basque area from the East through Europe and not through North Africa. However, the fact that we can trace some Basque lineages back to the Paleolithic does not support the generalized supposition that the present day Basque population is the best representative of Paleolithic Europeans. First of all, U8a haplotypes only represent 1% of the present day Basque maternal pool, therefore, a complex set of different mtDNA lineages with possible different histories are left unstudied. In addition, there is empiric evidence that Basques have received recent male gene flow from adjacent areas [31], and even possible maternal North African influences predating the Muslim Iberian invasion [32]. Furthermore, ancient DNA studies on Basque historic and prehistoric samples [33] have detected important mtDNA haplogroup frequency fluctuations along different periods. Definitively, like other European populations, Basques have also suffered migration and genetic drift effects throughout its long history.

## Methods

### Samples

DNA isolated from bucal swabs or blood samples from 211 autochthonous, unrelated Basques from the Iberian provinces were analyzed. Appropriate informed consent to anonymously use their data was obtained from all the individuals sampled.

### HVSI-II and RFLPs

Total DNA was PCR amplified as in Pinto et al [34], and directly sequenced for both complementary strands as detailed in Rando et al [35]. A sequence of 978 bp of the HVSI-II of the mtDNA control region, from position 15997 to 00408 [36] was determined and sorted into defined haplogroups [24]. To confirm this HVS-based haplogroup classification, all individuals assigned to a specific haplogroup were additionally tested by restriction analysis of the diagnostic coding region mutations proposed to unambiguously classify sequences into haplogroups [24].

### Complete mtDNA sequences

Four Basques (three U8a, one K1) and one Jordan (U8b) rare lineages belonging to the U/K haplogroup were fully sequenced. The complete mitochondrial DNAs (mtDNA) were amplified by PCR using primer pairs already described [19]. Amplified products were sequenced for both complementary strands with the Big Dye Terminator Cycle sequencing kit (Applied Biosystems). Sequencing reactions were analyzed on an Applied Biosystems 3100 DNA analyzer.

### Genetic analyses

Genetic diversity (p ± σ) was estimated as the average number of nucleotide differences between two sequences [37], using the HVSI region in the range from 16070 to 16365 nucleotide positions.

Phylogenetic relationships among complete mtDNA sequences, and among control region mtDNA sequences, were established using the reduced median network algorithm [11]. In addition to our five sequences, seven lineages were added: Dutch (Dut86; GenBank: DQ112821) [[38] and P. Shen, pers. comm], Spanish (Spa, GenBank: AY882392) and Italian (Ita, GenBank: AY882393) [20], 3 Finns (Fin; GenBank: AY339551, AY339552 and AY339553) [14] and British-Australian (BriT7; [T. Obayashi, M. Tanaka, pers. comm.]).

The presence of U8a was tested in published sequences by the 16146 16342 HVSI motif and/or the 73 282 HVSII motif, and U8b by the 16189 16234 motif. 19,133 Eurasian and 1,430 North-African published and unpublished HVSI/HVSII sequences were analyzed.

The average sequence divergence [16] for complete sequences was converted in time applying a mutation rate of 1.26 × 10^-8 ^[15].

Time estimations, based on control region mtDNA, were calculated as the mean divergence ρ [39] from inferred ancestral sequence types and converted into time by assuming that one transition within np 16090–16365 corresponds to 20,180 years [29].

### Accesion numbers

The five complete mitochondrial DNA sequences are registered in [GenBank: DQ200801, DQ200802, DQ200803, DQ200804, and DQ200805].

## Authors' contributions

We have distinguished the following principal steps in the work:

1) Conception and design

2) Collection of samples

3) Sequencing of the HVSI-II regions and RFLPs analysis

4) Complete mtDNA sequencing of rare lineages

5) Collection of data to assess the distribution of U8a/b haplogroups in Europe, Near-East, Asia and Africa

6) Phylogenetic reconstruction for HVSI, and complete mtDNA sequences

7) Discussion of the results obtained

8) To draft the manuscript (text, table, figures and additional files)

AMG participated in 1, 4, 5, 6, 7 and 8 steps. OG participated in 1,2, 3, 5, and 7 steps. JML participated in 1, 3, 4, 5, 7 and 8 steps. VMC participated in 1, 2, 4, 6, 7 and 8 steps. All authors read and approved the final manuscript.

## Supplementary Material

Additional File 1 European distribution of subhaplogroup U8a/1. File González_add1.xls have data about sample and percentage of U8a/1 haplogroup in different populations from Europe and North-Africa. Click here for file

Additional File 2 References cited in additional file 1. File González_add2.doc shows a list of references cited in additional file 1. Click here for file

## References

[B1] Barandiaran I, Barandiaran I, Marti B, Del Rincón MA, Maya JL (1998). El paleolítico y el mesolítico. Prehistoria de la Península Ibérica.

[B2] Cavalli-Sforza LL, Menozzi P, Piazza A (1994). The history and geography of human genes.

[B3] Bertranpetit J, Sala J, Calafell F, Underhill PA, Moral P, Comas D (1995). Human mitochondrial DNA variation and the origin of Basques. Ann Hum Genet.

[B4] Achilli A, Rengo C, Magri C, Battaglia V, Olivieri A, Scozzari R, Cruciani F, Zeviani M, Briem E, Carelli V, Moral P, Dugoujon JM, Roostalu U, Loogväli EL, Kivisild T, Bandelt HJ, Richards M, Villems R, Santachiara-Benerecetti AS, Semino O, Torroni A (2004). The molecular dissection of mtDNA haplogroup H confirms that the Franco-Cantabrian glacial refuge was a major source for the European gene pool. Am J Hum Genet.

[B5] Pereira L, Richards M, Goios A, Alonso A, Albarrán C, García O, Behar DM, Gölge M, Hatina J, Al-Gazali L, Bradley D, Macaulay V, Amorim A (2005). High-resolution mtDNA evidence for the late-glacial resettlement of Europe from an Iberian refugium. Genome Res.

[B6] Torroni A, Bandelt H-J, D'Urbano L, Lahermo P, Moral P, Sellitto D, Rengo C, Forster P, Savontaus M-L, Bonné-Tamir B, Scozzari R (1998). mtDNA analysis reveals a major late paleolithic population expansion from southwestern to northeastern Europe. Am J Hum Genet.

[B7] Torroni A, Bandelt H-J, Macaulay V, Richards M, Cruciani F, Rengo C, Martinez-Cabrera V, Villems R, Kivisild T, Metspalu E, Parik J, Tolk HV, Tambets K, Forster P, Karger B, Francalacci P, Rudan P, Janicijevic B, Rickards O, Savontaus ML, Huoponen K, Laitinen V, Koivumaki S, Sykes B, Hickey E, Novelletto A, Moral P, Sellitto D, Coppa A, Al-Zaheri N, Santachiara-Benerecetti AS, Semino O, Scozzari R (2001). A signal, from human mtDNA, of postglacial recolonization in Europe. Am J Hum Genet.

[B8] Loogväli E-L, Roostalu U, Malyarchuk BA, Derenko MV, Kivisild T, Metspalu E, Tambets K, Reidla M, Tolk H-V, Parik J, Pennarun E, Laos S, Lunkina A, Golubenko M, Barac L, Pericic M, Balanovsky PO, Gusar V, Khusnutdinova EK, Stepanov V, Puzyrev V, Rudan P, Balanovska EV, Grechanina E, Richard C, Moisan J-P, Chaventré A, Anagnou NP, Pappa KI, Michalodimitrakis EN, Claustres M, Gölge M, Mikerezi I, Usanga E, Villems R (2004). Disuniting uniformity: A pied cladistic canvas of mtDNA haplogroup H in Eurasia. Mol Biol Evol.

[B9] Harpending HC, Eller E, Kato M, Takahata N (1999). Human diversity and its history. The Biology of biodiversity.

[B10] Richards M, Macaulay V (2001). The mitochondrial gene tree comes of age. Am J Hum Genet.

[B11] Bandelt H-J, Forster P, Röhl A (1999). Median-joining networks for inferring intraspecific phylogenies. Mol Biol Evol.

[B12] Palanichamy MG, Sun C, Agrawal S, Bandelt H-J, Kong Q-P, Khan F, Wang C-Y, Chaudhuri TK, Palla V, Zhang Y-P (2004). Phylogeny of mitochondrial DNA macrohaplogroup N in India, based on complete sequencing: Implications for the peopling of South Asia. Am J Hum Genet.

[B13] Herrnstadt C, Elson JL, Fahy E, Preston G, Turnbull DM, Anderson C, Ghosh SS, Olefsky JM, Beal MF, Davis RE, Howell N (2002). Reduced-median-network analysis of complete mitochondrial DNA coding-region sequences for the major African, Asian, and European haplogroups. Am J Hum Genet.

[B14] Finnilä S, Lehtonen MS, Majamaa K (2001). Phylogenetic network for European mtDNA. Am J Hum Genet.

[B15] Mishmar D, Ruiz-Pesini E, Golik P, Macaulay V, Clark AG, Hosseini S, Brandon M, Easley K, Chen E, Brown MD, Sukernik RI, Olckers A, Wallace DC (2003). Natural selection shaped regional mtDNA variation in humans. Proc Natl Acad Sci USA.

[B16] Saillard J, Forster P, Lynnerup N, Bandelt H-J, Nurby S (2000). MtDNA variation among Greenland Eskimos: the edge of the Beringian expansion. Am J Hum Genet.

[B17] Quintana-Murci L, Chaix R, Wells RS, Behar DM, Sayar H, Scozzari R, Rengo C, Al-Zahery N, Semino O, Santachiara-Benerecetti AS, Coppa A, Ayub Q, Mohyuddin A, Tyler-Smith C, Qasim Mehdi S, Torroni A, McElreavey K (2004). Where west meets east: The complex mtDNA landscape of the southwest and central Asian corridor. Am J Hum Genet.

[B18] Harpending HC, Rogers AR (2000). Genetic perspectives on human origins and differentiation. Ann Rev Genomics Hum Genet.

[B19] Maca-Meyer N, González AM, Larruga JM, Flores C, Cabrera VM (2001). Major genomic mitochondrial lineages delineate early human expansions. BMC Genet.

[B20] Achilli A, Rengo C, Battaglia V, Pala M, Olivieri A, Fornarino S, Magri C, Scozzari R, Babudri N, Santachiara-Benerecetti AS, Bandelt HJ, Semino O, Torroni A (2005). Saami and Berbers–An unexpected mitochondrial DNA link. Am J Hum Genet.

[B21] Tanaka M, Cabrera VM, González AM, Larruga JM, Takeyasu T, Fuku N, Guo L-J, Hirose R, Fujita Y, Kurata M, Shinoda K-i, Umetsu K, Yamada Y, Oshida Y, Sato Y, Hattori N, Mizuno Y, Arai Y, Hirose N, Ohta S, Ogawa O, Tanaka Y, Kawamori R, Shamoto-Nagai M, Maruyama W, Shimokata H, Suzuki R, Shimodaira H (2004). Mitochondrial genome variation in eastern Asia and the peopling of Japan. Genome Res.

[B22] Dubut V, Chollet L, Murail P, Cartault F, Beraud-Colomb E, Serre M, Mogentale-Profizi N (2003). MtDNA polymorphisms in five French groups: importance of regional sampling. Eur J Hum Genet.

[B23] Bermisheva MA, Tambets K, Villems R, Khusnutdinova EK (2002). Diversity of mitochondrial DNA haplogroups in ethnic populations of the Volga-Ural region. Mol Biol.

[B24] Richards M, Macaulay V, Hickey E, Vega E, Sykes B, Guida V, Rengo C, Rengo C, Sellito D, Cruciani F, Kivisild T, Villems R, Thomas M, Rychkov S, Rychkov O, Rychkov Y, Gölge M, Dimitrov D, Hill E, Bradley D, Romano V, Cali F, Vona G, Demaine A, Papiha S, Triantaphyllidis C, Stefanescu G, Hatina J, Belledi M, Di Rienzo A, Novelleto A, Oppenheim A, Norby S, Al-Zaheri N, Santachiara-Benerecetti S, Scozzari R, Torroni A, Bandelt H-J (2000). Tracing European founder lineages in the Near Eastern mtDNA pool. Am J Hum Genet.

[B25] Thomas MG, Weale ME, Jones AL, Richards M, Skorecki K, Torroni A, Scozzari R, Gratrix F, Tarekegn A, Wilson JF, Capelli C, Bradman N, Goldstein DB (2002). Founding mothers of Jewish communities: geographically separated Jewish groups were independently founded by very few female ancestors. Am J Hum Genet.

[B26] Cherni L, Loueslati BY, Pereira L, Ennafaâ H, Amorim A, El Gaaied ABA (2005). Female gene pools of Berber and Arab neighboring communities in Central Tunisia: Microstructure of mtDNA variation in North Africa. Hum Biol.

[B27] Metspalu M, Kivisild T, Metspalu E, Parik J, Hudjashov G, Kaldma K, Serk P, Karmín M, Behar DM, Gilbert MT, Endicott P, Mastana S, Papiha SS, Skorecki K, Torroni A, Villems R (2004). Most of the extant mtDNA boundaries in south and southwest Asia were likely shaped during the initial settlement of Eurasia by anatomically modern human. BMC Genet.

[B28] Bandelt H-J, Forster P, Sykes BC, Richards MB (1995). Mitochondrial portraits of human populations using median networks. Genetics.

[B29] Forster P, Harding R, Torroni A, Bandelt H-J (1996). Origin and evolution of Native American mtDNA variation: a reappraisal. Am J Hum Genet.

[B30] Gamble C (1999). The Paleolithic societies of Europe.

[B31] Hurles ME, Veitia R, Arroyo E, Armenteros M, Bertranpetit J, Pérez-Lezaun A, Bosch E, Shlumukova M, Cambon-Thamsen A, McElreavey K, López de Munain A, Rohl A, Wilson LJ, Singh L, Pandya A, Santos FR, Tyler-Smith C, Jobling MA (1999). Recent male-mediated gene flow over a linguistic barrier in Iberia, suggested by analysis of a Y-chromosomal DNA polymorphism. Am J Hum Genet.

[B32] Alzualde A, Izaguirre N, Alonso S, Alonso A, Albarrán C, Azkarate A, De la Rúa C (2005). Insights into the "isolation" of the Basques: mtDNA lineages from the historical site of Aldaieta (6)^th^–7^th ^c. AD). Am J Phys Anthropol in press.

[B33] Alzualde A, Izagirre N, Alonso S, Alonso A, de la Rúa C (2005). Temporal mitochondrial DNA variation in the Basque country: Influence of post-Neolithic events. Ann Hum Genet.

[B34] Pinto F, González AM, Hernández M, Larruga JM, Cabrera VM (1996). Genetic relationship between the Canary Islanders and their African and Spanish ancestors inferred from mitochondrial DNA sequences. Ann Hum Genet.

[B35] Rando JC, Pinto F, González AM, Hernández M, Larruga JM, Cabrera VM, Bandelt H-J (1998). Mitochondrial DNA analysis of northwest African populations reveals genetic exchanges with European, near-eastern, and sub-Saharan populations. Ann Hum Genet.

[B36] Anderson S, Bankier AT, Barrell BG, de Bruijn MH, Coulson AR, Drouin J, Eperon IC, Nierlich DP, Roe BA, Sanger F, Schreier PH, Smith AJ, Staden R, Young IG (1981). Sequence and organisation of the human mitochondrial genome. Nature.

[B37] Nei M (1987). Molecular evolutionary genetics.

[B38] Kivisild T, Shen P, Wall DP, Do B, Sung R, Davis KK, Passarino G, Underhill PA, Scharfe C, Torroni A, Scozzari R, Modiano D, Coppa A, de Knjiff P, Feldman MW, Cavalli-Sforza LL, Oefner PJ (2006). The role of selection in the evolution of human mitochondrial. Genetics.

[B39] Morral N, Bertranpetit J, Estivill X, Nunes V, Casals T, Giménez J, Reis A, Varon-Mateeva R, Macek M, Kalaydjieva L, Angelicheva D, Dancheva R, Romeo G, Russo MP, Garnerone S, Restagno G, Ferrari M, Magnani C, Claustres M, Desgeoges M, Schwartz M, Dallapiccola B, Novelli G, Ferec C, de Arce M, Nemeti M, Kere J, Anvret M, Dahl N, Kadasi L (1994). The origin of the major cystic fibrosis mutation (Δ F508) in European populations. Nat Genet.

