# Supplementary material for: The mitochondrial lineage U8a reveals a Paleolithic settlement in the Basque country
Source: BMC Genomics. 2006 May 23;7:124. doi: 10.1186/1471-2164-7-124 (PMC1523212; doi:10.1186/1471-2164-7-124)
Supplement: Additional File 2 — References cited in additional file 1. File González_add2.doc shows a list of references cited in additional file 1. [file 1471-2164-7-124-S2.doc]

References used in appendix I

1.- Baasner A., Schäfer C., Junge A., Madea B. 1998. Polymorphic sites in human mtDNA control region sequences: population data and maternal inheritance. Foren Sci Int 98:169-178

2.- Baasner A., Madea B. 2000. Sequence polymorphisms of the mitochondrial DNA control region in 100 German caucasians. J Forensic Sci. 45:1343-1348

3.- Babalini C., Martinez-Labarga C., Tolk H.V., Kivisild T., Giampaolo R., Tarsi T., Contini I., Barac L., Janicijevic B., Martinovic Klaric I., Pericic M., Sujoldzic A., Villems R., Biondi G., Rudan P., Rickards O. 2005. The population history of the Croatian linguistic minority of Molise (southern Italy): a maternal view. Eur J Hum Genet. 13(8):902-912

4.- Baranov P.O., Babenko V.N., Ivanova A.V., Kobzev V.F., Romashchenko A.G., Voevoda M.I. 1999. Characteristics of the mitochondrial genome of russian germans.Genetika. 35(2):249-254

5.- Barbujani G., Stenico M., Excoffier L., Nigro L. 1996. Mitochondrial DNA sequence variation across linguistic and geographic boundaries in Italy. Hum Biol. 68 201-215

6.- Behar, D.M., Hammer, M.F., Garrigan, D., Villems R., Bonne-Tamir, B., Richards, M., Gurwitz, D., Rosengarten D., Kaplan, M., Pergola, S.D. Quintana-Murci, L., Skorecki K. 2004. MtDNA evidence for a genetic bottleneck in the early history of the Ashkenazi Jewish population. Eur. J. Hum. Genet.12(5):355-365

7.- Belledi M., Poloni E.S., Casalotti R., Conterio F., Mikerezi I., Tagliavini J., Excoffier L. 2000. Maternal and paternal lineages in Albania and the genetic structure of Indo-European populations. Eur J Hum Genet. 8:480-486

8.- Belyaeva O., Bermisheva M., Khrunin A., Slominsky P., Bebyakova N., Khusnutdinova E., Mikulich A., Limborska S. 2003. Mitochondrial DNA variations in Russian and Belorussian populations. Hum Biol. 75:647-660

9.- Bermisheva M.A., Tambets K., Villems R., Khusnutdinova E.K. 2002. Diversity of mitochondrial DNA haplogroups in ethnic populations of the Volga-Ural region. Mol Biol. 36,6: 802-812

10.- Bermisheva M.A., Kutuev I.A., Korshunova T.Y., Dubova N.A., Villems R., Khusnutdinova E.K. 2004. Phylogeografic analysis of mitochondrial DNA Nogays: the high level of mixture of maternal lineages from Eastern and Western. Mol Biol 38 (4):617-624

11.- Bertranpetit J., Sala J., Calafell F., Underhill P.A., Moral P., Comas D. 1995. Human mitochondrial DNA variation and the origin of the Basques. Ann Hum Genet 59:63-81

12.-Bini C., Ceccardi S., Luiselli D., Ferri G., Pelotti S., Colalongo C., Falconi M., Pappalardo G. 2003. Different Informativeness of the three hypervariable mitochondrial DNA regions in the population of Bologna (Italy).Forensic Sci Int. 135(1):48-52

13.- Brakez Z., Bosch E., Izaabel H., Akhayat O., Comas D., Bertranpetit J., Calafell F. 2001. Human mitochondrial DNA sequence variation in the Moroccan population of the Souss area. Ann Hum Biol 28(3):295-307

14.- Calafell F., Underhill P., Tolun A., Angelicheva D., Kalaydjeva L. 1996. From Asia to Europe: mitochondrial DNA sequence variability in Bulgarians and Turks. Ann Hum Genet 60:35-49

15.- Cali F., Le Roux M.G., D'Anna R., Flugy A., De Leo G., Chiavetta V., Ayala G.F., Romano V.J. 2001. MtDNA control region and RFLP data for Sicily and France. Legal Med.;114(4-5):229-231

16.- Cherni L., Loueslati B.Y., Pereira L., Ennafaâ H., Amorim A., El Gaaied A.B.A. 2005. Female gene pools of Berber and Arab neighboring communities in Central Tunisia: Microstructure of mtDNA variation in North Africa. Hum. Biol. 77: 61-70

17.- Comas D., Calafell F., Mateu E., Perez-Lezaun A., Bertranpetit J. 1996. Geographic variation in human mitochondrial DNA control region sequence: the population history of Turkey and its relationship to the European populations. Molec Biol Evol 13:1067-1077

18.- Comas D., Calafell F., Bendukidze N., Fananas L., Bertranpetit J. 2000. Georgian and kurd mtDNA sequence analysis shows a lack of correlation between languages and female genetic lineages. Am J Phys Anthropol. 112(1):5-16

19.- Corte-Real H.B., Macaulay V.A., Richards M.B., Hariti G., Issad M.S., Cambon-Thomsen A., Papiha S., Bertranpetit J., Sykes B.C. 1996. Genetic diversity in the Iberian Peninsula determined from mitochondrial sequence analysis. Ann Hum Genet 60:331-350

20.- Crespillo M., Luque J.A., Paredes M., Fernández R., Ramírez E., Valverde J.L. 2000. Mitochondrial DNA sequences for 118 individuals from northeastern Spain. Int. J. Legal. Med. 114:130-132

21.- Di Benedetto G., Erguven A., Stenico M., Castri L., Bertorelle G., Togan I., Barbujani G. 2001. DNA diversity and population admixture in Anatolia. Am J Phys Anthropol. 115:144-156

22.- Di Rienzo A., Wilson A.C. 1991. Branching pattern in the evolutionary tree for human mitochondrial DNA. Proc Nat Acad Sci, USA. 88:1597-1601

23.- Dimo-Simonin N., Grange F., Taroni .F, Brandt-Casadevall C., Mangin P. 2000. Forensic evaluation of mtDNA in a population from south west Switzerland. Int J Legal Med. 113:89-97

24.- Dubut V., Chollet L., Murail P., Cartault F., Beraud-Colomb E., Serre M., Mogentale-Profizi N. 2004. DNA polymorphisms in five French groups: importance of regional sampling. Eur J Hum Genet. 12(4):293-300

25.- Dupuy B.M., Olaisen B. 1996. mtDNA sequences in the Norwegian Saami and main populations. Adv Forensic Haemogenet 6 :23-25

26.- Fadhlaoui-Zid K., Plaza S., Calafell F., Ben Amor M., Comas D., Bennamar El Gaaied A. 2004. Mitochondrial DNA heterogeneity in Tunisian Berbers. Ann Hum Genet 68(3):222-233

27.- Forster P., Cali F., Rohl A., Metspalu E., D'Anna R., Mirisola M., De Leo G., Flugy A., Salerno A., Ayala G., Kouvatsi A., Villems R., Romano V. 2002. Continental and subcontinental distributions of mtDNA control region types Int J Legal Med. 116(2):99-108

28.- Francalacci P., Bertranpetit J., Calafell F., Underhill, P. 1996. Sequence Diversity of the Control Region of Mitochondrial DNA in Tuscany and Its Implications for the Peopling of Europe. Am J Phys Anthropol. 100:443-460

29.- Fraumene C., Petretto E., Pirastu A.A.M. 2003. Striking differentiation of sub-populations within a genetically homogeneous isolate (Ogliastra) in Sardinia as revealed by mtDNA analysis. Hum Genet. 114:1-10

30.- González A.M., Brehm A., Pérez J.A., Maca-Meyer N., Flores C., Cabrera V.M. 2003. Mitochondrial DNA affinities at the Atlantic fringe of Europe.Am J Phys Anthropol. 120(4):391-404

31.- González A.M., Cabrera V.M., Larruga J.M., Tounkara A., Noumsi G., Thomas B.N, Moulds J.M. 2005. Mitochondrial DNA variation in Mauritania and Mali and their genetic relationship to other western Africa populations. Ann Hum Genet, in press

32.- Helgason A., Siguroardottir S., Gulcher J.R., Ward R., Stefansson K. 2000. DNA and the origin of the Icelanders: deciphering signals of recent population history. Am J Hum Genet. 66(3):999-1016

33.- Helgason A., Hickey E., Goodacre S., Bosnes V., Stefansson K., Ward R., Sykes B. 2001. MtDna and the islands of the North Atlantic: estimating the proportions of Norse and Gaelic ancestry. Am J Hum Genet. 68(3):723-737

34.- Hofmann S., Jaksch M., Bezold R., Mertens S., Aholt S., Paprotta A., Gerbitz K.D. 1997. Population genetics and disease susceptibility: characterization of central European haplogroups by mtDNA gene mutations, correlation with D loop variants and association with disease. Hum Mol Genet. 6(11):1835-1846

35.- Jorde L.B., Bamshad M.J., Watkins W.S., Zenger R., Fraley A.E., Krakowiak P.A., Carpenter K.D., Soodyall H., Jenkins T., Rogers A.R. 1995. Origins and affinities of modern humans: a comparison of mitochondrial and nuclear genetic data.  Am J Hum Genet  57:523-538

36.- Kasperaviciute D., Kucinskas V., Stoneking M. 2004. Y chromosome and mitochondrial DNA variation in Lithuanians. Ann Hum Genet. 68:438-452

37.- Kittles R.A., Bergen A.W., Urbanek M., Virkkunen M., Linnoila M., Goldman D., Long J.C. 1999. Autosomal, mitochondrial, and Y chromosome DNA variation in Finland: evidence for a male-specific bottleneck. Am J Phys Anthropol. 108(4):381-399

38.- Kouvatsi A., Karaiskou N., Apostolidis A., Kirmizidis G. 2001. Mitochondrial DNA sequence variation in Greeks. Hum Biol. 73(6):855-869

39.- Krings M., Halim Salem A., Bauer K., Geisert H., Malek A.K., Chaix L., Simon C., Welsby D.1999. MtDNA analysis of Nile valley populations: A genetic corridor or a barrier to migration? Am J Hum Genet 64(4):1166-1176

40.- Lahermo P., Sajantila A., Sistonen P., Lukka M., Aula P., Peltonen L., Savontaus M.L. 1996. The genetic relationship between the Finns and the Finnish Saami (Lapps): analysis of nuclear DNA and mtDNA. Am J Hum Genet 58:1309-1322

41.- Larruga J.M., Díez F., Pinto F.M., Flores C., González A.M. 2001. Mitochondrial DNA characterisation of European isolates: the Maragatos from Spain. Eur J Hum Genet. 9(9):708-716

42.- López-Soto M., Sanz P. 2000. Polimorfismos de ADN mitocondrial en individuos residentes en Andalucía y Extremadura. Cuad Med Foren. 20: 17-24

43.- Lutz S., Weisser H-J., Heizmann J., Pollak S. 1998. Location and frequency of polymorphic positions in the mtDNA control region of individuals from Germany.  Int J Legal Med 111:67-77

44.- Maca-Meyer N., Sánchez-Velasco P., Flores C., Larruga J.M., González A.M., Oterino A., Leyva-Cobian F. 2003. Y chromosome and mitochondrial DNA characterization of Pasiegos, a human isolate from Cantabria (Spain). Ann Hum Genet. 67:329-339

45.- Macaulay, V., Richards, M., Hickey, E., Vega, E., Cruciani, F., Guida, V., Scozzari, R., Bonné-Tamir, B., Sykes, B., Torroni, A. 1999. The emerging tree of West Eurasian mtDNAs: a synthesis of control-region sequences and RFLPs. Am. J. Hum. Genet. 64: 232-249

46.- Malyarchuk B.A., Derenko M.V. 2001. Mitochondrial DNA variability in Russians and Ukrainians: implication to the origin of the Eastern Slavs. Ann Hum Genet. 65:63-78

47.- Malyarchuk B.A., Grzybowski T., Derenko M.V., Czarny J., Wozniak M., Miscicka-Sliwka D. 2002. Mitochondrial DNA variability in Poles and Russians. Ann. Hum. Genet. 66:261-283

48.- Malyarchuk B.A., Grzybowski T., Derenko M.V., Czarny J., Drobnic K., Miscicka-Sliwka D. 2003. Mitochondrial DNA variability in Bosnians and Slovenians. Ann Hum Genet. 67:412-425

49.- Malyarchuk B.A., Derenko M.V., Grzybowski T., Lunkina A., Czarny J., Rychkov S., Morozova I., Denisova G., Miscicka-Sliwka D. 2004.Differentiation of mitochondrial DNA and Y chromosomes in Russian populations. Hum Biol. 76(6):877-900

50.- Martínez-Jarreta B., Prades A., Calafell F., Budowle B. 2000. Mitochondrial DNA HVI and HVII variation in a north-east Spanish population.Forensic Sci. 45(5):1162-1163

51.- McEvoy B., Richards M., Forster P., Bradley D.G. 2004. The Longue Duree of genetic ancestry: multiple genetic marker systems and Celtic origins on the Atlantic facade of Europe. Am J Hum Genet. 75(4):693-702.

52.- Meinila M., Finnila S., Majamaa K. 2001. Evidence for mtDNA admixture between the Finns and the Saami. Hum Hered.52(3):160-170

53.- Mergen H., Öner R., Öner C. 2004. Mitochondrial DNA sequence variation in the Anatolian Peninsula (Turkey). J Genet 83(1):101-109

54.- Miller K.W.P. 1996. Molecular Genetic Analysis of Human Populations in Orkney and the North Atlantic Region. University of Cambridge.

55.- Mogentale-Profizi N., Chollet L., Stevanovitch A., Dubut V., Poggi C., Pradie M.P., Spadoni J.L., Gilles A., Beraud-Colomb E. 2001. Mitochondrial DNA sequence diversity in two groups of Italian Veneto speakers from Veneto. Ann Hum Genet. 65:153-166

56.- Nasidze I., Stoneking M. 2001. Mitochondrial DNA variation and language replacements in the Caucasus. Proc. R. Soc. Lond. 268:1197-1206

57.- Nasidze I., Ling E.S., Quinque D., Dupanloup I., Cordaux R., Rychkov S., Naumova O., Zhukova O., Sarraf-Zadegan N., Naderi G.A., Asgary S., Sardas S., Farhud D.D., Sarkisian T., Asadov C., Kerimov A., Stoneking M. 2004. Mitochondrial DNA and Y-chromosome variation in the Caucasus. Ann Hum Genet. 68: 205-221

58.- Nasidze I., Quinque D., Dupanloup I., Rychkov S., Naumova O., Zhukova O., Stoneking M. 2004. Genetic evidence concerning the origins of South and North Ossetians. Ann Hum Genet. 68:588-599

59.- Opdal S.H., Rognum T.O., Vege A., Stave A.K., Dupuy B.M., Egeland T. 1998. Increased number of substitutions in the D-loop of mitochondrial DNA in the sudden infant death syndrome. Acta Paediatr. 87(10):1039-1044

60.- Orekhov V., Poltoraus A., Zhivotovsky L.A., Spitsyn V., Ivanov P., Yankovsky N. 1999. Mitochondrial DNA sequence diversity in Russians.FEBS Lett. 445(1):197-201

61.- Parson W., Parsons T.J., Scheithauer R., Holland M.M.. 1998. Population data for 101 Austrian Caucasian mitochondrial DNA d-loop sequences: application of mtDNA sequence analysis to a forensic case. Int J Legal Med. 111:124-32

62.- Passarino G., Cavalleri G.L., Lin A.A., Cavalli-Sforza L.L., Borresen-Dale A.L., Underhill P.A. 2002. Different genetic components in the Norwegian population revealed by the analysis of mtDNA and Y chromosome polymorphisms. Eur J Hum Genet. 10(9):521-529

63.- Pereira L., Cunha C., Amorim A. 2004. Predicting sampling saturation of mtDNA haplotypes: an application to an enlarged Portuguese database. Int J Legal Med. 118(3):132-136

64.- Pfeiffer H., Brinkmann B., Huhne J., Rolf B., Morris AA., Steighner R., Holland M.M., Forster P. 1999. Expanding the forensic German mitochondrial DNA control region database: genetic diversity as a function of sample size and microgeography. Int J Legal Med. 112:291-298

65.- Pfeiffer H., Forster P., Ortmann C., Brinkmann B. 2001. The results of an mtDNA study of 1,200 inhabitants of a German village in comparison to other Caucasian databases and its relevance for forensic casework. Int J Legal Med. 114(3):169-172

66.- Picornell A., Gomez-Barbeito L., Tomas C., Castro J.A., Ramon M.M.. 2005. Mitochondrial DNA HVRI variation in Balearic populations.Am J Phys Anthropol. 128(1):119-130

67.- Piercy R., Sullivan K., Benson N., Gill P. 1993. The application of mitochondrial DNA typing to the study of white Caucasian genetic identification. Int. J. Leg. Med. 106:85-90

68.- Plaza S., Calafell F., Helal A., Bouzerna N., Lefranc G., Bertranpetit J., Comas D. 2003. Joining the pillars of Hercules: mtDNA sequences show multidirectional gene flow in the western Mediterranean. Ann Hum Genet. 67(4):312-328

69.- Poetsch M., Wittig H., Krause D., Lignits E. 2003. Mitochondrial diversity of a northeast German population sample. Foren Sci Int. 137(2-3):125-132

70.- Pult I., Sajantila A., Simanainem J., Georgiev O., Schaffner W., Paabo S. 1994. Mitochondrial DNA sequences from Switzerland reveal striking homogeneity of European populations. Biol. Chen. Hoppe Seyler. 375:837-840

71.- Quintana-Murci L., Chaix R., Wells R.S., Behar D.M., Sayar H., Scozzari R., Rengo C., Al-Zahery N., Semino O., Santachiara-Benerecetti A.S., Coppa A., Ayub Q., Mohyuddin A., Tyler-Smith C., Qasim Mehdi S., Torroni A., McElreavey K. 2004. Where west meets east: the complex mtDNA landscape of the southwest and Central Asian corridor. Am J Hum Genet. 74(5):827-845

72.- Rando J.C., Pinto F., González A.M., Hernández M., Larruga J.M., Cabrera V.M., Bandelt H.J. 1998. Mitochondrial DNA analysis of northwest African populations reveals genetic exchanges with European, near-eastern, and sub-Saharan populations. Ann Hum Genet 62:531-550

73.- Richards M., Corte-Real H., Forster P., Macaulay V., Wilkinson-Herbots H., Demaine A., Papiha S., Hedges R., Bandelt H.J., Sykes B. 1996. Paleolithic and Neolithic lineages in the European mitochondrial gene pool. Am J Hum Genet 59:185-203

74.- Richards M., Macaulay V., Hickey E., Vega E., Sykes B., Guida V., Rengo C., Sellitto D., Cruciani F., Kivisild T., Villems R., Thomas M., Rychkov S., Rychkov O., Rychkov Y., Golge M., Dimitrov D., Hill E., Bradley D., Romano V., Cali F., Vona G., Demaine A., Papiha S., Triantaphyllidis C., Stefanescu G., Hatina J., Belledi M., Di Rienzo A., Novelletto A., Oppenheim A., Norby S., Al-Zaheri N., Santachiara-Benerecetti S., Scozari R., Torroni A., Bandelt H.J. 2000. Tracing European founder lineages in the Near Eastern mtDNA pool. Am J Hum Genet. 67(5):1251-1276

75.- Rickards O., Martínez-Labarga C., Casalotti R., Castellana G., Tunzi Sisto A.M., Mallegni F. 2000. Mitochondrial DNA variability in extinct and extant populations of Sicily and Southern Italy. In Archaeogenetics: DNA and the population prehistory of Europe. Edited by Colin Renfrew& Katie Boyle McDonald Institute for Archaeological Research. University of Cambridge. Cambridge

76.- Rousselet F., Mangin P. 1998. Mitochondrial DNA polymorphisms: a study of 50 French Caucasian individuals and application to forensic casework. Int J Legal Med. 111(6):292-298

77.- Saillard J., Evseeva I., Tranebjaerg L., Norby S. 2000. Mitochondrial DNA diversity among Nenets. In Archaeogenetics: DNA and the population prehistory of Europe. Edited by Colin Renfrew& Katie Boyle McDonald Institute for Archaeological Research. University of Cambridge. Cambridge

78.- Sajantila A., Lahermo P., Anttinen T., Lukka M., Cistonen P., Savontaus M.-L., Aula P., Beckman L., Tranebjaerg L., Gedde-Dahl T., Issel-Tarver L., DiRienzo A., Paabo S. 1995. Genes and languages in Europe: an analysis of mitochondrial lineages. Genome Res 5:42-52

79.- Sajantila A., Salem A.H., Savolainen P., Bauer K., Gierig C., Paabo S. 1996. Paternal and maternal DNA lineages reveal a bottleneck in the founding of the Finnish population. Proc Natl Acad Sci U S A. 93(21):12035-12039

80.- Salas A., Comas D., Lareu M.V., Bertranpetit J., Carracedo A. 1998. mtDNA analysis of the Galician population: a genetic edge of European variation. Eur J Hum Genet. 6(4):365-375

81.- Salas A, Lareu V, Calafell F, Bertranpetit J, Carracedo A. 2000. mtDNA hypervariable region II (HVII) sequences in human evolution studies. Eur J Hum Genet. 8:964-974

82.- Stenico M., Nigro L., Bertorelle G., Calafell F., Capitanio M., Corrain C., Barbujani G. 1996. High Mitochondrial DNA Sequence Diversity in Linguistic Isolates of the Alps. Am J Hum Genet. 59:1363-1375

83.- Stevanovitch A., Gilles A., Bouzaid E., Kefi R., Paris F., Gayraud R.P., Spadoni J.L., El-Chenawi. 2003. Mitochondrial DNA Sequence diversity in a sedentary population from Egypt. Ann Hum Genet 68:23-39

84.- Tagliabracci A., Turchi C., Buscemi L., Sassaroli C. 2001. Polymorphism of the mitochondrial DNA control region in Italians Int J Legal Med. 114(4-5):224-228

85.- Thomas M.G., Weale M.E., Jones A..L, Richards M., Smith A., Redhead N., Torroni A., Scozzari R., Gratrix F., Tarekegn A., Wilson J.F., Capelli .C, Bradman N., Goldstein D.B. 2002. Founding mothers of Jewish communities: geographically separated Jewish groups were independently founded by very few female ancestors. Am J Hum Genet. 70(6):1411-1420

86.- Tolk H.V., Barac L., Pericic M., Klaric I.M., Janicijevic B., Campbell H., Rudan I., Kivisild T., Villems R., Rudan P. 2001. The evidence of mtDNA haplogroup F in a European population and its ethnohistoric implications. Eur J Hum Genet. 9(9):717-723

87.- Troesch V., Clisson I., Petraud M., Ludes B., Jaeck-Brignou E. 2003. Polymorphism of D-loop mitochondrial DNA: study of HVI and HV2 regions in unrelated individuals living in the East of France. Progress in Forensic Genetics 9: 529-534

88.- Vanecek T., Vorel F., Sip M. 2004. Mitochondrial DNA D-loop hypervariable regions: Czech population data. Int. J. Legal Med 118:14-18

89.- Varesi L. , Memmí M., Cristofari M.-C., Mameli G.E. , Caló C.M., Vona G. 2000. Mitochondrial control-region sequence variation in the Corsican population, France. Am J Hum Biol 12:339-351

90.- Verginelli F., Donati F., Coia V., Boschi I., Palmirotta R., Battista P., Costantini R.M., Destro-Bisol G. 2003. Variation of the hypervariable region-1 of mitochondrial DNA in central-eastern Italy. J Forensic Sci. 48(2):443-444

91.- Vernesi C., Fuselli S., Castri L., Bertorelle G., Barbujani G. 2002. Mitochondrial diversity in linguistic isolates of the Alps: a reappraisal. Hum Biol. 74(5):725-730

92.- Villems R., Adojaan M., Kivisild T., Metspalu E., Parik, J., Pielberg, G., Rootsi, S., Tambets, K., Tolk H-V. 1998. Reconstruction of maternal lineales of Finno-Ugric speaking people and some remarks on their paternal inheritance. In The roots of people and languages in Northern Eurasia I. Julku, K. and Wiik, K., eds. pp 180-200, Tartu

93.- Vona G., Ghiani M.E., Caló C.M., Vacca L., Memmí M., Vares, L. 2001. Mitochondrial DNA variation sequence análisis in Sicily. Am J Hum Biol. 13:576-589

94.- Watson E., Foster P., Richards M., Bandelt H.J. 1997. Mitochondrial Footprints of Human Expansions in Africa. Am J Hum Genet 61:691-704

95.- Zupanic Pajnic I., Balazic J., Komel R. 2004. Sequence polymorphism of the mtDNA control region in the Slovenian population. Int. J. Legal Med. 2004. 118:1-4

96.- Unpublished data
